# Anxiety related disorders in adolescents in the United Arab Emirates: a population based cross-sectional study

**DOI:** 10.1186/s12887-020-02155-0

**Published:** 2020-05-25

**Authors:** Nabeel Al-Yateem, Wegdan Bani issa, Rachel C. Rossiter, Arwa Al-Shujairi, Hadia Radwan, Manal Awad, Randa Fakhry, Ibrahim Mahmoud

**Affiliations:** 1 University of Sharjah, Sharjah, UAE; 2 School of Nursing, Midwifery & Indigenous Health, Faculty of Science, Charles Sturt University, Leeds Parade, Orange, New South Wales Australia; 3 Research Institute of Medical and Health Sciences, Sharjah, UAE

**Keywords:** Adolescents, Anxiety, School, United Arab Emirates

## Abstract

**Background:**

Anxiety disorders are common among children and adolescents. However, there is a paucity of up-to-date data on the prevalence and correlates of anxiety-related disorders among children and adolescents in the United Arab Emirates (UAE).

**Methods:**

We conducted a cross sectional study to determine the prevalence of specific anxiety-related disorders (e.g., generalized anxiety disorder, panic disorder, separation anxiety, social anxiety) in the UAE, and identify correlations between these disorders and adolescents’ demographic variables. Participants were 968 adolescents aged 13–18 years attending secondary schools across the UAE. Convenience sampling was used to recruit participants. We collected demographic information and data about participants’ anxiety levels. Anxiety was assessed using the Arabic and English versions of the Screen for Child Anxiety Related Disorders scale. Univariate analyses (independent sample *t*-tests and analysis of variance) were performed to evaluate factors affecting participants’ anxiety scores. Chi-square tests were used to compare factors associated with anxiety disorders.

**Results:**

Participants’ mean age was 16 ± 1.8 years, and 65.8% were female. The overall prevalence of anxiety disorders was 28%, with this being significantly higher in girls (33.6%) than boys (17.2%) (*p* < 0.0001). Participants aged < 16 years had higher generalized anxiety, separation anxiety, and social anxiety scores compared with those aged ≥16 years (*p* ≤ 0.05). Those from households with a maid had significantly higher generalized anxiety, panic disorder, separation anxiety, and significant school avoidance scores than those without a maid (*p* ≤ 0.05). In addition, participants from middle and low economic backgrounds had higher separation anxiety scores compared with children from high economic backgrounds (*p* ≤ 0.05). The multivariate analysis showed the main associated factors with anxiety were gender (being female, *p* < 0.001) and caregiver (other than mother and father together, *p* < 0.001).

**Conclusions:**

We found a high incidence of anxiety-related disorders among school-aged adolescents in the UAE, with girls being more affected than boys. This suggests that age-appropriate initiatives are urgently needed to reduce the high rate of anxiety-related disorders. It may also be necessary to further investigate the two main associated factors with anxiety identified in this study (being female and non-parental caregivers).

## Background

Anxiety is a normal human emotion characterized by various responses (e.g., behavioral, affective, and cognitive) to perceived threat [1]. However, anxiety can be considered excessive or pathological when such responses cause significant distress or are out of proportion to the perceived source of stress [1]. The World Health Organization (WHO) reported the number of people with anxiety or depression increased by almost 50% between 1990 and 2013 [2], with around 10% of the world’s population affected by these disorders.

Anxiety disorders are common among children and adolescents [1, 3, 4]. Reported rates of anxiety among children and adolescents were 31.9% in the United States (age: 13–18 years) [5], 26.41% in Spain (age: 8–17 years) [6], 22.5% in Chile (age: 4–18 years) [7], 21.9% in Iran [4], and 36.7% in India (secondary school children) [8]. Anxiety disorders that remain undetected and untreated in childhood and adolescence may affect well-being in adulthood, which challenges earlier views that high levels of anxiety are developmentally normal [9, 10].

Current diagnostic frameworks identify several anxiety disorders that commonly occur during childhood and adolescence, including generalized anxiety disorder (GAD), panic disorder, social anxiety disorder, and significant school refusal/avoidance disorder [11–13]. Although there are differing perspectives on the etiology of anxiety in childhood, multiple factors (e.g., the child’s temperament and characteristics, genetic factors, environmental factors) are thought to contribute to the development of anxiety disorders among children and adolescents [14]. Specific risk factors include adverse family experiences (e.g., marital conflict, death of a parent), school stressors (e.g., bullying), abuse (emotional, physical, or sexual), maternal substance abuse, and parental mental health [15, 16]. Parental characteristics (e.g., education level, unemployment) or living without parents may also contribute to the risk for anxiety disorders among young people [17]. Anxiety disorders are also reported to be more prevalent in girls and among children with comorbidities or chronic conditions (e.g., diabetes) [14, 18–20]. Although there is some evidence that heritability (i.e., anxiety runs in families) may have a role in anxiety, there is debate as to whether this can be explained by modeling of anxious behaviors within a family [1, 21, 22]. However, anxiety disorders may involve complex interactions between a child’s unique characteristics and their environment [23].

Although anxiety disorders among children and adolescents are common, they remain distressing and impairing for the child/adolescent and the family. In particular, school and social functioning is lower in children with anxiety disorders compared with children without such disorders [24]. Anxiety disorders may also interfere with a young person’s social communication, peer relationships, schooling, and family life [19, 25]. Despite anxiety being common and debilitating in children and adolescents, it frequently remains unidentified and untreated [24]. For example, a previous study found teachers had limited sensitivity to variations in students’ levels of anxiety symptoms, and often struggled to identify students that required targeted interventions or additional classroom support [26]. Research conducted in the United Arab Emirates (UAE) reported that correct identification of mental health problems and accurate identification of appropriate evidence-based interventions for affected children was limited among healthcare professionals [27, 28]. This was attributed to low levels of mental health literacy among respondents, combined with religious and cultural factors that potentially affected their interventions.

The UAE is a progressive, highly developed, and stable country, but is surrounded by countries experiencing political and economic instability. Approximately 80–90% of the UAE population is expatriates and immigrants [29]. Challenges for these people include separation from their families and loved ones, financial hardships, and potentially having witnessed violence or atrocities back home. In addition, the UAE has a large percentage of young people with developmental needs that may predispose them to anxiety in adulthood, especially those with chronic conditions [27]. The UAE also has specific environmental and cultural risk factors that contribute to mental health problems, particularly among young people [28, 30]. These risk factors include large family units and consanguineous marriages, which are common in the UAE and the wider Arab world [31].

Much of the epidemiological research on anxiety disorders in children and adolescents has been conducted in Western settings [32]. Data on the prevalence, comorbidity, and predictors of anxiety disorders among children and adolescents are scarce in the Middle East, including in the UAE. Previous UAE-based studies in this area date back to 1998 [33] and 2004 [34], and more up-to-date data are required. It is of particular concern that many social, emotional and behavioral problems among young children are not identified during pediatric healthcare contacts [35], which suggests a preventive approach is necessary. An initial national epidemiological study is needed to clarify the extent of the problem in the UAE, increase awareness of this issue, and inform further interventional studies. This study aimed to determine the prevalence of specific anxiety-related disorders (i.e., GAD, panic disorder, separation anxiety, social anxiety, and significant school avoidance), and identify correlations between these disorders and adolescents’ demographic variables.

## Methods

### Study design

This study was part of a larger study that used a cross-sectional, correlational design to collect a comprehensive dataset from adolescents attending schools across the UAE. Accessing participants from schools enabled inclusion of young people from a range of cultural and socioeconomic backgrounds. The dataset covered adolescents’ demographic data and variables that were previously reported to be related to adolescents’ health and well-being, including obesity, nutritional status, physical activity, dental health, smoking status, and anxiety. The present study focused on anxiety-related disorders, to determine its prevalence and correlates with adolescents’ demographic variables.

### Study population

The target population was adolescents aged 13–18 years attending public or private secondary schools across the UAE. To be eligible for participation, students needed to be literate in either Arabic or English and provide written parental consent.

### Sampling method

We initially planned to use a two-stage clustered randomized sampling approach, with stage one being identifying the schools for inclusion and stage two identifying a randomized sample of students for recruitment. However, accessing accurate information for all schools in the UAE and enrolments in those schools proved unachievable. Therefore, convenience sampling was used to recruit schools and students. First, we compiled a list of private and public secondary schools offering intermediate and high school education using available data from the seven emirates (Sharjah, Dubai, Abu Dhabi, Ajman, Ras-Al-Khaimah, Al Fujairah, and Um Al Quwain). The principals of these schools were contacted to seek initial approval for their school to participate in this study. Schools that gave approval were then visited and provided with full information about the study. Following formal approval from the schools, school principals provided access to classes from grades 9–12 based on class schedules and students’ availability. Students were given information packs and consent forms to take home. Those who returned consent forms signed by their parent/guardian and signed an assent form themselves were enrolled in this study.

### Sample size

As this examination of anxiety was undertaken as one component of a larger multi-variate study as mentioned earlier, it was necessary to select a main variable for sample size calculation. Obesity was selected as the main variable as it was reported to be correlated with key variables identified for the overall study (i.e., nutritional status, physical activity levels, and smoking). Previous research undertaken in the UAE reported the prevalence of overweight and obesity among UAE adolescents was approximately 40% [36]. Therefore, a total sample size of 1124 students was needed for this study, using a 3% margin of error at a 95% confidence interval and significance level of 0.05.

### Data collection process

A total of 1100 students from selected classrooms in various schools around the UAE who met the inclusion criteria were enrolled in this study. Students completed a questionnaire that collected demographic information and data about anxiety levels. The questionnaire was administered by research assistants in students’ classrooms, meaning students received instructions and clarification immediately as needed. Data collection and entry took place from May 2016 to May 2018.

### Data collection instrument

To measure anxiety levels, we used the Arabic and English versions of the Screen for Child Anxiety Related Disorders (SCARED) scale, developed by Birmaher and colleagues [37]. The validity and reliability of the SCARED scale have been assessed using item and factor analyses [37]. The scale has four domains of anxiety: panic/somatic, separation anxiety, generalized anxiety, and school phobia. The scale comprises five factors, specifically: panic/somatic, generalized anxiety, separation anxiety, social phobia, and school phobia. The overall scale and all subscales have good internal consistency and discriminant validity within anxiety disorders and between anxiety, depressive, and disruptive disorders [38]. The scale has been translated and used with differing cultural and linguistic populations.

An Arabic version of the SCARED scale (A-SCARED) has been developed, with validity and reliability established in Arabic speaking Lebanon [39] and Saudi Arabia [40]. The general reliability score of the A-SCARED was reported as α = 0.91 [39]. The concurrent validity of the A-SCARED was established by administering it with the Arabic Strengths and Difficulties Questionnaire; the two scales showed good correlation (r = 0.70, *p* = 0.001). The A-SCARED contains 41 questions rated on a three-point Likert scale: “Not True/Hardly Ever True,” “Somewhat True/Sometimes True,” and “Very True/Often True.”

### Statistical analysis

Descriptive analyses were performed using means and standard deviations (SD) for continuous variables, and frequencies and percentages for categorical variables. Univariate analyses including independent sample *t*-tests and analysis of variance were performed to evaluate factors affecting participants’ anxiety scores. Chi-square tests were used to compare proportions of anxiety disorders between boys and girls. We considered *p*-values ≤0.05 as statistically significant for all analyses, and all tests were two tailed. Finally, logistic regression adjusted for confounding factors was used to identify the strongest predictors of anxiety. All analyses were performed using SPSS version 25 (IBM Corp, New York, USA).

### Ethical considerations

Ethics approval was obtained from University of Sharjah Research Ethics Committee and from the UAE Ministry of Health and Prevention. The research team strictly adhered to principles of confidentiality and privacy. Coding was used to ensure participants’ confidentiality, with these codes used to replace participants’ personal data in all documentation. Data were only accessible to the research team, and all data used in publications related to this study were de-identified.

## Results

In total, 968 questionnaires were completed. Participants’ mean age was 16 years (SD 1.8 years), and almost two-thirds were female (65.8%). The majority of participants were local Emiratis (61.8%). Approximately 80% of participants were from three Emirates: Sharjah (40%), Dubai (20%), and Ajman (19.8%). Table 1 presents participants’ demographic characteristics.

**Table 1:** Participants’ characteristics (*N* = 968)

	Characteristic	n (%)
**Gender**	Boys	331 (34.2)
	Girls	637 (65.8)
**Nationality**	Local Emirati	598 (61.8)
	Expatriate	370 (38.2)
**Age, years**	Mean ± SD	15.9 ± 1.6
**Age groups, years**	< 16	353 (36.5)
	≥16	615 (63.5)
**School**	Private	441 (45.6)
	Public	527 (54.4)
**Mother’s employment status**	Employed	258 (26.8)
	Not employed	705 (73.2)
**Economic status**	High	370 (38.2)
	Middle	563 (58.2)
	Low	35 (3.6)
**Maid**	Yes	588 (60.7)
	No	380 (39.3)
**Caregiver**	Mother	327 (54.7)
	Father	41 (6.7)
	Mother and father	203 (34)
	Other	27 (4.5)
	Missing	370 (38)
**Medical condition**	Yes	173 (27.5)
	No	456 (72.5)

SD, Standard deviation

Evaluation of total anxiety disorder scores showed the mean score was 25.3 ± 14.3 for girls and 18.9 ± 12 for boys (*p* < 0.0001). Girls had higher mean scores for all types of anxiety disorders (Table 2). Participants aged < 16 years had higher anxiety, generalized anxiety, separation anxiety, and social anxiety scores compared with those aged ≥16 years (*p* ≤ 0.05). Participants from households with a maid had showed significantly higher scores for anxiety, panic disorder, separation disorder, and significant school avoidance than those without a maid (p ≤ 0.05). Participants whose main caregiver was someone other than their mother/father showed significantly higher scores for anxiety (30.2 ± 17.1), panic disorder (9.0 ± 6.3), social anxiety (6.03 ± 4.4), generalized anxiety (6.6 ± 4.6), and school avoidance (3.5 ± 2.8) compared with those cared for by their mother only, father only, and both mother and father (*p* < 0.05). There were no significant differences in anxiety scores by mother’s employment status and economic status (*p* > 0.05). However, participants from low and middle economic backgrounds showed higher scores for separation anxiety compared with those from high economic backgrounds (*p* ≤ 0.05). Table 2 presents analysis of factors affecting anxiety disorder scores.

**Table 2:** Bivariate analysis of factors affecting anxiety disorder scores in adolescents, by anxiety type, mean (standard deviation), (*N* = 968)

		Anxiety	Panic	Generalized	Separation	Social	School avoidance
**Gender**	Boys	18.9 (12)*	4.4 (4.1)*	4.5 (3.6)*	3.8 (2.8)*	4.2 (3.3)*	1.9 (1.8)*
	Girls	25.3 (14.3)*	6.8 (5.4)*	6.2 (4.3)*	4.8 (3.1)*	5.2 (3.5)*	2.3 (1.8)*
**Nationality**	Emirate	23.3 (13.9)	6.1 (5.2)	5.4 (4.1)*	4.6 (3.1)*	4.9 (3.4)	2.3 (1.8)*
	Expatriate	22.7 (13.9)	5.8 (5.1)	6 (4.3)*	4.1 (2.9)*	4.8 (3.6)	2 (1.8)*
**Age groups, years**	< 16	24.6 (13.6)*	6.3 (5.2)	6.2 (4.2)*	4.7 (3.1)*	5.2 (3.4)*	2.1 (1.9)
	≥16	22.3 (14)*	5.8 (5.1)	5.3 (4.1)*	4.3 (3)*	4.7 (3.4)*	2.2 (1.8)
**School**	Private	24 (14.4)	6.2 (5.3)	6.3 (4.4)*	4.4 (3.2)	5 (3.6)	2 (1.9)*
	Public	22.3 (13.4)	5.8 (5)	5 (3.9)*	4.5 (2.9)	4.7 (3.3)	2.3 (1.8)*
**Mother’s employment status**	Employed	23.1 (12.9)	5.8 (4.6)	5.9 (4)	4.4 (2.8)	4.8 (3.3)	2.1 (1.8)
	Not employed	23.1 (14.2)	6 (5.3)	5.5 (4.2)	4.4 (3.1)	4.9 (3.5)	2.2 (1.8)
**Economic status**	High	22.5 (13.8)	5.8 (5.2)	5.7 (4.2)	4.3 (3.1)*	4.7 (3.4)	2.1 (1.9)
	Middle	23.2 (13.6)	6 (5)	5.6 (4.1)	4.5 (3)*	4.9 (3.4)	2.3 (1.8)
	Low	28.1 (17.5)	7.4 (6.4)	6.2 (4.6)	5.7 (4.1)**	6.1 (3.4)	2.7 (2.1)
**Maid**	Yes	24 (14)*	6.3 (5.2)*	5.7 (4.2)	4.6 (3.1)*	5 (3.4)	2.4 (1.8)*
	No	21.6 (13.7)*	5.5 (5)*	5.4 (4.1)	4.1 (3)*	4.6 (3.5)	2 (1.9)*
**Caregiver**	Mother only	24.6 (14)*	6.5 (5.2)*	5.6 (4.0)*	4.7 (3.0)	5.1 (3.4)*	2.4 (1.9)*
	Father only	24.1 (13.6)*	6.5 (4.9)*	5.7 (4.0)*	4.1 (3.2)	5.2 (3.5)*	2.2 (1.4)*
	Mother and father	20 (12.1)*	4.9 (4.3)*	4.0 (3.2)*	4.3 (2.8)	4.3 (3.2)*	2.2 (1.4)*
	Other	30.2 (17.1)*	9.0 (6.3)*	6.6 (4.6)*	4.9 (3.2)	6.03 (4.4)*	3.5 (2.8)*
**Medical condition**	Yes	25.4 (14.5)*	6.7 (5.4)*	6.1 (4.9)*	4.9 (3.2)*	5.2 (3.7)	2.6 (1.7)*
	No	22.2 (13.4)*	5.7 (5)*	5.2 (3.9)*	4.3 (2.9)*	4.7 (3.3)	2.2 (1.8)*

* *p* ≤ .05

The overall prevalence of anxiety disorders was 28% (Table 3), with the prevalence being significantly higher in girls (33.6%) than in boys (17.2%) (*p* < 0.0001). The multivariate analysis (Table 4) showed that gender and caregiver were the main associated factors for anxiety. Girls were more likely to develop anxiety symptoms than boys (odds ratio [OR] 2.34, 95% CI: 1.45–3.73). Adolescents who were cared for by both their mother and father were less likely to develop anxiety compared with adolescents who were raised by someone other than their mother and father (OR 0.30, 95% CI: 0.12–0.72).

**Table 3:** Distribution of anxiety disorders (score > 30) by gender, based on chi-square tests, n (%)

Anxiety disorders	Total	Boys	Girls	p-value
Anxiety (score > 30)	271 (28)	57 (17.2)	214 (33.6)	< 0.0001
Generalized (score > 9)	209 (21.6)	45 (13.6)	164 (25.7)	< 0.0001
Panic (score > 7)	359 (37.1)	83 (25.1)	276 (43.3)	< 0.0001
Separation (score > 5)	440 (45.5)	124 (37.5)	316 (49.6)	< 0.0001
Social (score > 8)	194 (20)	49 (14.8)	145 (22.8)	0.003
Significant school avoidance (score > 3)	353 (36.5)	96 (29)	257 (40.3)	0.001

**Table 4:** Logistic regression model of anxiety in adolescents with gender, caregiver, medical condition, maid, and age as predictors (*N* = 936)

	Categories	Adjusted OR	95% CI	p-value
**Gender**	Male	1	–	–
	Female	2.34	1.45–3.73	< 0.001
**Caregiver**	Other	1	–	–
	Father	0.81	0.29–2.25	0.255
	Mother	0.50	0.22–1.16	0.332
	Mother and Father	0.30	0.12–0.72	< 0.001
**Medical condition**	No	1	–	
	Yes	1.32	0.89–1.97	0.169
**Maid**	No	1	–	–
	Yes	0.96	0.65–1.41	0.773
**Age, years**	≥16	1	–	–
	< 16	0.76	0.49–1.17	0.207

OR, odds ratio; CI, confidence interval

## Discussion

The UAE is actively developing its healthcare system and other services (e.g., education and tourism) with the aim of being at the forefront of the world in these areas. In the health sector, the UAE Vision 2021 specifies the objective of building and providing world-class healthcare for the population and tourists [41]. The UAE has also identified mental health as among the top five priorities for healthcare services that need to be addressed. The present national epidemiological study was therefore essential to shed light on the prevalence of anxiety disorders among adolescents in the UAE and allow comparisons with regional and international data. This will help to inform policy and service decisions on future interventions, studies, and initiatives.

This study focused on adolescents, as evidence suggests the majority of mental disorders begin before the age of 14 years. Our study population was also aligned with the demographic composition of the UAE population, which is mainly composed of children, adolescents, and young adults. UAE statistics indicate that a significant proportion of this age group suffer from chronic illnesses [42]. This suggests a significant proportion of the population may be vulnerable to mental health disorders [27], and highlights the urgency of raising awareness and knowledge among healthcare providers and developing informed national initiatives.

This study found the overall prevalence of anxiety disorders among school-aged adolescents in the UAE to be 28%, which was higher than rates in other countries. The prevalence of anxiety-related disorders among adolescents in this study was significantly higher than the worldwide-pooled prevalence of 6.5% reported by Polanczyk et al. [3], which drew on data from 27 countries in multiple regions. In addition, although the prevalence of anxiety in our study was lower compared with the United States (31.9%) [5] and similar to Spain (26.41%) [6], it was higher than rates reported in Chile (22.5%) [7] and Iran (21.9%) [4]. An Indian study reported much higher rates, although that study included participants in the late adolescence developmental stage [8].

The gender differences in the prevalence of anxiety in this study were supported by previous literature, which reported a higher incidence of anxiety-related disorders among girls [14, 24, 43, 44]. We also found that the prevalence of anxiety-related disorders in children younger than age 16 years was higher than that among children older than 16 years. This may indicate that while anxiety onset is in early childhood, some children may navigate their way out of anxiety through internal anxiety management resources and appropriate support from their caregivers. An interesting and unique finding from this study was that children who reported a maid as their primary caregiver had significantly higher scores for anxiety, panic disorder, separation disorder, and significant school avoidance disorder compared with those cared for by their mother only, father only, and both mother and father. The lowest levels of anxiety were observed in children who were cared for by both parents. This finding may be of particular importance and relevance to the UAE and neighboring countries, where there is a high reliance on maids and domestic helpers for household duties and child care [45].

Our multiple logistic regression analysis of associated factors with developing anxiety among adolescents in the UAE revealed that the main associated factors with anxiety were gender and caregiver. Girls were more likely to develop anxiety symptoms than boys, and those who were raised with a high contribution from a maid were more likely to have anxiety compared with those raised by both their mother and father. The cultural context of the UAE may explain these two factors, especially the over-reliance on domestic help for raising children [46]. In addition, despite government efforts to support women, there remains strict traditional rules that may place extra pressure on girls and women, and limit their life choices (e.g., study, career, and travel). Such limitations may place females at higher risk for developing anxiety compared with males.

Anxiety disorders have also been associated with headaches, sleep difficulties, stuttering and other speech disorders [47–49], and interfere with a young person’s social, school, and family life [19, 26]. This means it is important for these disorders to be identified and treated early. The WHO [2] suggests that investing in early treatment for depression and anxiety leads to a fourfold return. A number of effective treatments are available for anxiety disorders, including psychological therapies (especially cognitive behavioral therapy), education for children and their parents, and pharmacological therapy (as needed), with a combined or multi-modal approach often considered most effective [14]. Online or Internet-based psychological therapies [50, 51] may also be effective for adolescents with anxiety disorders.

### Study limitations

This study gathered data from most Emirates in the UAE, but could not gather data from Abu Dhabi because of access issues. As Abu Dhabi is the main and largest Emirate, this may reduce the representativeness of the study. In addition, it is acknowledged that the UAE has recently become a hub that has received many refugees fleeing from troubled countries in the region. This factor might have affected the data collected and prevalence of anxiety-related disorders observed in this study. We did not collect specific data to verify the background of any such participants.

## Conclusions

This study revealed an alarmingly high incidence of anxiety-related disorders among adolescents in the UAE (28%). Immediate local initiatives are needed to address this problem and reduce the high rate of anxiety-related disorders. Initiatives to reduce anxiety in the UAE should consider the two main associated factors identified in this study (being female and non-parental caregivers). Targeted support is also needed for girls to prevent, manage, or reduce anxiety. Strategies may also need to be considered to reduce the reliance on domestic helpers in raising children and provide appropriate support for parents to raise children themselves.

## Data Availability

The datasets used and/or analyzed during the present study are available from the corresponding author on reasonable request.

## Abbreviations

UAE: United Arab Emirates
WHO: World Health Organization
GAD: Generalized Anxiety Disorders
SCARED: Scale for Child Anxiety Related Disorders
A-SCARED: Arabic Scale for Child Anxiety Related Disorders
